# Optimised expression and purification of RBP4 and preparation of anti‐RBP4 monoclonal antibody

**DOI:** 10.1002/2211-5463.13349

**Published:** 2021-12-24

**Authors:** Hui Li, Xiao He, Sha Wen, Lichao Yang, Qiuli Chen, Yasi Li, Shiping Huang, Xuejing Huang, Fengjie Wan, Min He

**Affiliations:** ^1^ School of Public Health Guangxi Medical University Nanning China; ^2^ School of Public Health Guilin Medical School China; ^3^ Laboratory Animal Center Guangxi Medical University Nanning China; ^4^ Department of Public Health Sciences College of Medicine Pennsylvania State University Hershey PA USA; ^5^ Ministry of Education Key Laboratory of High‐Incidence‐Tumor Prevention & Treatment (Guangxi Medical University Nanning China

**Keywords:** immunohistochemical analysis, monoclonal antibody, RBP4, recombinant protein

## Abstract

The expression level of retinol‐binding protein 4 (RBP4) protein is closely related to liver damage and plays an important role in the diagnosis and prognosis of cancer. However, the preparation of anti‐RBP4 mAb or exploration on the application of anti‐RBP4 mAb has not been reported thus far. In the present study, we constructed a pET30a‐RBP4 recombinant vector, used *E. coli* BL21 (DE3) as the vector to express the RBP4 recombinant protein and prepared anti‐RBP4 mAb using hybridoma technology. We performed immunohistochemical analysis on hepatocellular carcinoma (HCC) and adjacent tissues by using this anti‐RBP4 mAb. In addition to the high‐purity RBP4 recombinant protein, we successfully developed the anti‐RBP4 mAb with high affinity and specificity; it binds to natural RBP4 protein and is suitable for immunohistochemical analysis.

## Graphical abstract

The expression level of RBP4 protein is closely related to liver damage and plays an important role in the diagnosis and prognosis of tumours. In this study, we successfully developed an anti‐RBP4 mAb with high affinity and specificity; it is bound to natural RBP4 protein and is suitable for immunohistochemical analysis.

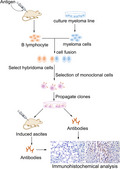

**Abbreviations**
DAB: diaminobenzidine
DE3: *E. coli* BL21
ECL: enhanced chemiluminescence
HCC: hepatocellular carcinoma
iELISA: indirect enzyme‐linked immunosorbent sandwich assay
IHC: immunohistochemistry
IOD: integrated optical density
IPTG: isopropyl‐β‐d‐thiogalactoside
KEGG: kyoto encyclopedia of genes and genomes
NEK2‐His: never in mitosis gene‐A‐related expressed kinase 2‐His
RBP4: retinol‐binding protein 4
SAA4‐His: serum amyloid A‐4‐His
SDS/PAGE: sodium dodecyl sulphate/polyacrylamide gel electrophoresis
SHBG‐His: sex hormone‐binding globulin‐His
TCGA: the cancer genome atlas

Retinol‐binding protein 4 (RBP4) is a member of the lipocalin super family and is mainly secreted by the liver. RBP4 is secreted into the blood to combine with retinol, playing a crucial role in the transport of retinol and vitamin A [1]. In 2005, QIN Y et al. found that RBP4 acted as an adipose factor and participated in insulin resistance. Moreover, many studies have revealed that RBP4 is closely related to insulin‐resistant type 2 diabetes [2], cardiovascular disease [3], metabolic syndrome [4, 5] and other diseases. In recent years, studies have reported that RBP4 is associated with cancer, and it has a remarkable effect in promoting the migration and proliferation of ovarian cancer cells. The underlying molecular mechanism of RBP4 involves the activation of the RhoA/Rock1 pathway and the expression of CyclinD1. It may represent a potential target for the treatment of obese cancer patients [6]. High serum RBP4 levels are associated with an increased risk of colon adenoma and pancreatic cancer [7, 8]. The RBP4‐STRA6 pathway drives the maintenance of cancer stem cells and mediates the occurrence of colon cancer induced by a high‐fat diet [9]. With the deepening of research, RBP4 has been shown to be involved in the occurrence and development of liver diseases [10]. Relevant studies have indicated that serum RBP4 concentration is negatively correlated with the severity of disease in early HCV patients, and as the serum RBP4 concentration decreases, the degree of liver fibrosis increases proportionally [11, 12]. Moritoshi et al. [13] found that the low expression of RBP4 in HCC tissues was closely related to the development of HCC. In the early stage of this study, transcriptomics sequencing and proteomics technology analysis demonstrated that RBP4 had low expression in the serum of patients with HCC, suggesting that it may become a diagnostic marker for HCC. Researchers have constructed an enzyme‐linked immunosorbent double antibody sandwich assay kit based on anti‐RBP4 mouse IgA mAb to facilitate the detection of RBP4 [14]. However, reports on the production process of optimised expression and purification of RBP4 protein or the production of high‐specificity and high‐affinity anti‐RBP4 mAb are few. In addition, commercial RBP4 antibodies and kits vary widely amongst different manufacturers and different batches; thus, the production of high specificity anti‐RBP4 mAb is required.

We expressed the RBP4 recombinant protein using the prokaryotic expression system. After purification, the recombinant protein was immunised to BALB/c mice to prepare anti‐RBP4 mAb. Anti‐RBP4 mAb could be used to detect the RBP4 expression level by immunohistochemistry (IHC) on the basis of the expression level of RBP4 in HCC tissues found on The Cancer Genome Atlas (TCGA) database. Therefore, the proposed mAb has advantages of high affinity and specificity for RBP4 protein, thereby providing insights in advancing auxiliary examination of related diseases.

## Materials and methods

### Cell lines, plasmid, experimental animals, main reagents and instruments

Normal human liver cell line HL‐7702, HCC cell hep3B, HCC cell huh7 and SP2/0 mouse myeloma cells were purchased from the Shanghai Academy of Biological Sciences and Cell Collection and Chinese Academy of Sciences. Strain DH5α competent cells and *E. coli* BL21 (DE3) were purchased from Beijing Quanshijin Company. The plasmid pET‐30a was donated by the School of Life Science and Technology of Guangxi University. SPF‐grade 4‐week‐old female BALB/c mice, weighing 14 g‐17 g, were sourced from Shanghai Slack Laboratory Animal Co., Ltd. [SCXK (Shanghai) 2017‐0005], with certificate number: 20170005000441. All mice were housed in specific pathogen‐free facilities and cared for in Laboratory Animal Center of Guangxi Medical University [SCXK (Gui) 2020‐003]. Sterile surgery was conducted in the Experimental Animal Centre of Guangxi Medical University. All animal experiments had been approved by the Laboratory Animal Welfare and Ethics Committee of Guangxi Medical University (NO. 201911011) and performed in accordance with national ethics regulations. In the experimental design, we had achieved the minimum necessary amount by optimising the experimental scheme. In the process of the experiment, we used a relatively mild injection technique and appropriate amount of adjuvant to reduce the serious side effects of animals and reduce the pain of the animals. Other materials purchased include the following: total RNA extraction and transcription kit, 10× T4 DNA ligase (Takara, Japan), *RBP4* gene primers (Beijing Ruiboxingke, China), plasmid extraction kit (Omega Bio‐Tek, USA), Hind III and BamH I enzymes (Promega, USA), 2× Fast Pfu PCR enzyme (Beijing full gold, China), Ni‐Agarose Resin (China Kangwei Century, China), SDS/PAGE protein gel reagents and BCA kits (Beyotime biological company, China), Freund’s complete adjuvant, Freund’s incomplete adjuvant, HRP‐labelled goat anti‐mouse secondary antibody (Thermo, USA), Bovine Serum Albumin (BSA) (Solebold, China), gradient RT‐PCR instrument (Bio‐Rad, USA), gel image analyser (BIO‐BAD, USA), ultrasonic cell disruptor (Ningbo Xinzhi Company, China), western blot electrophoresis imaging system (Thermo, USA), EVOS FL automatic cell imager (Thermo, USA) and HCC tissue sample chip (Shanghai Xinchao, China).

### Cell culture

HL‐7702 and Huh7 were cultured in DMEM containing 10% FBS, and Hep3B was cultured in MEM containing 10% FBS. Mouse myeloma cell line SP2/0 was cultured in RPMI‐1640 medium containing 10% FBS, hybridoma cell lines were cultured in RPMI‐1640 medium containing 20% FBS and 20% HAT, and monoclonal hybridoma cell lines were cultured in RPMI‐1640 medium containing 20% FBS and 20% HT. All the cells were incubated at 37 °C in 5% CO2.

### Construction of recombinant plasmid

The amino acid sequence of RBP4 protein was obtained from Kyoto Encyclopedia of Genes and Genomes (KEGG) database, and the position of its signal peptide was analysed by SignalP5.0. The antigenic index of RBP4 protein was predicted by DNAstart software, and the appropriate sequence was selected for expression. The *RBP4* primer sequences were AAAGGATCCGAGCGC GACTGCCGAGTGAG (forward) and GGGAAGCTTCTACAAAAGGTTTCTTTCTG (reverse). Total RNA of a normal human liver cell line HL‐7702 was extracted as raw material, and RBP4 gene was obtained by RT‐PCR amplification. The reaction conditions are as follows: 95 °C 3 min, 95 °C 30 s, 61 °C 30 s, 72 °C 90 s, and 35 cycles; 4 °C infinite cycle. A small amount of pET‐30a plasmid was extracted with the kit, and the pET‐30a plasmid and *RBP4* gene were digested with BamH I enzyme and Hind III enzyme, respectively. The ligated vector was transformed into competent DH5α and plated onto LB kanamycin plates. The next day, a single colony was picked from the plate and cultured in LB, and the recombinant plasmid was identified by restriction enzyme digestion with BamH I and Hind III.

### Recombinant protein expression, condition optimisation and purification

The pET30a‐*RBP4* plasmid and pET30a empty plasmid were divided into two groups, namely the IPTG‐induced group and the uninduced group. The IPTG‐induced group was added with isopropyl‐β‐d‐thiogalactoside (IPTG) to a final concentration of 1.0 mmol·L^−1^, and the expression was induced at 37 °C for 4 h. The bacterial pellet was broken by ultrasonic disruption, and the bacterial supernatants and the precipitates were collected separately. The expression form of the recombinant protein was identified by 12% sodium dodecyl sulphate/polyacrylamide gel electrophoresis (SDS/PAGE). According to these protein expression steps, different induction temperatures (18, 28, 37 and 42 °C) and different induction IPTG concentrations (0, 0.2, 0.4, 0.6, 0.8 and 1 mm) were considered to determine the optimal expression conditions. The supernatant protein solution was added to a chromatography column containing 2 mL Ni agarose gel packing and incubated overnight at 4 °C on a shaker to purify the recombinant protein. Proteins were eluted with 150 mm imidazole elution concentration, which was increased from 50 mm to 200 mm to determine the optimal elution condition, and the elution peaks containing a large amount of RBP4 protein were collected. This solution was purified by 250 mmol·L^−1^ KCl staining. Finally, the RBP4 recombinant protein containing His tag was confirmed by western blot using anti‐His‐tag antibody.

### Mice immunisation and serum titre detection by indirect enzyme‐linked immunosorbent sandwich assay (iELISA)

The first dose was mixed with the same volume of complete Freund’s adjuvant at a dose of 150 μg·mouse^−1^, and the BALB/c female mice were immunised with multiple subcutaneous injections at an interval of 14 days, using recombinant RBP4 protein at a concentration of 1 μg·μL^−1^ as the immunogen. For the second immunisation, the dose of 80 μg·mouse^−1^ was mixed with the same volume of Freund’s incomplete adjuvant, and the interval was 2 weeks. The dose of the third and fourth immunisations, identical to the second, was given at 2‐week interval. Seven days after the fourth immunisation, the iELISA was used to detect the serum titre of mouse tail vein blood. In summary, the 96‐well microplate was coated with 100 μL RBP4 recombinant protein (2 μg·mL^−1^) at 4 °C overnight. The positive serum and negative serum samples were diluted in a threefold ratio, from 1 : 1000 to 1 : 729,000, and the 0.1% BSA solution was used as a blank control. The HRP‐IgG goat anti‐mouse (1 : 10,000) was added and incubated at 37 °C for 30 min. Under dark conditions, the substrate colour solution was added with 100 μL·well^−1^. Finally, the reaction was stopped by 2 m sulphuric acid, and the OD_450nm_ value was measured. The serum dilution corresponding to positive serum (*P*)/negative serum (*N*) value ≥ 2.1 was used as the serum titre of immunised mice. In addition, immunised mice with serum titre greater than 1 : 10,000 were selected for the next cell fusion.

### Cell fusion

Mice with serum titre greater than 1 : 10,000 were selected for cell fusion. Three days after booster immunisation of mice, spleen cells and SP2/0 cells are fused at a ratio of 2 : 10 under the concentration of 50% PEG. After fusion, the hybridoma cells were cultured in 1/3 HAT selection medium. After 9 days, the hybridoma cell supernatants were determined by iELISA. The positive hybridoma cell lines were selected and subjected to three subclonal screening by the limiting dilution method.

### mAb preparation and identification

BALB/c mice were sensitised intraperitoneally with 500 μL of paraffin. After 7 days, the 1 × 10^7^ monoclonal hybridoma cells were injected into the mice abdominal cavity to prepare ascites. The monoclonal antibodies were purified by the rProtein G affinity chromatography column. The mouse mAb isotyping (IgA, IgM, IgG1, IgG2a, IgG2b and IgG3) detection kit was used to detect mAb isotype. The iELISA method was used to determine the antibody affinity constant. According to the checkerboard square method, the RBP4 recombinant protein was coated on the 96‐well microplate at the concentration of 4, 2, 1 and 0.5 μg·mL^−1^, and the selected mAb was serially diluted with a double dilution ratio (1 : 1000‐1 : 1,024,000). The calculation method of affinity constant Ka was the same as that reported in the previous study [15]. Recombinant RBP4‐His protein, recombinant sex hormone‐binding globulin‐His (SHBG‐His) protein, recombinant serum amyloid A‐4‐His (SAA4‐His) protein, recombinant never in mitosis gene‐A related expressed kinase 2‐His (NEK2‐His) protein and BSA albumin were coated on the 96‐well microplate at a concentration of 2 μg·mL^−1^, and the sample diluent was used as a negative control. According to the iELISA method, the anti‐RBP4 mAb was diluted at 1 : 20,000 and then tested. HCC cell line hep3B, HCC cell huh7 total protein, SHBG‐His recombinant protein, RBP4‐His recombinant protein and RBP4 protein standard (R&D) were electrophoresed in 12% SDS/PAGE gel and transferred into the PVDF membrane. The membrane was incubated with purified anti‐RBP4 mAb (1 : 10,000) overnight at 4 °C. HRP‐IgG goat anti‐mouse (1 : 10,000) was added and incubated for 60 min at room temperature, the membrane was washed with TBST buffer for three times and then developed with enhanced chemiluminescence (ECL).

### Immunohistochemical assays

The tissue chip contains tissue samples from 10 hepatocellular carcinoma (HCC) patients and the corresponding adjacent tissues. In accordance with the improved nuclear grading scheme of the Edmondson and Steiner system, all tissue samples were pathologically diagnosed as hepatocellular carcinoma, and the histological grade was categorised as grade I ~ II (*n* = 1), II (*n* = 4), II ~ III (*n* = 2) and III (*n* = 3). A summary of the pathological characteristics and the patient characteristics is presented in Table 1. The expression of RBP4 was analysed in 10 pairs of HCC tissues and adjacent non‐tumorous liver tissues based on the standard immunohistochemistry procedures. The sample was incubated with the anti‐RBP4 mAb (1 : 1500) for 60 min at 37 °C. The staining of the sample was performed using the diaminobenzidine (DAB) solution at room temperature for 3–5 min and then counterstained with haematoxylin. Other immunohistochemical assay procedures were the same as that reported in the study [16]. image‐pro Plus 6.0 software was used for semi‐quantification of the staining. In summary, three areas of each sample were captured randomly at 40× the magnification of EVOS FL automatic cell imager, and the measurement parameter was the integrated optical density (IOD). The parameter setting referred to the method of Li Gang et al. [17], and then, the values of log IOD were counted.

**Table 1:** Relationships between RBP4 expression and clinicopathological variables of HCC. NA, No value.

Characteristics	*n*	RBP4 (lg IOD)	
		Mean ± SD	*P*‐value
Age (years)			
≤ 50	5	1.67 ± 1.72	0.552
> 50	5	0.91 ± 1.94	
Edmondson grade			
I‐II	5	1.19 ± 1.88	0.857
III	5	1.41 ± 1.87	
Liver metastasis			
Yes	0	NA	NA
No	10	1.30 ± 1.77	
Tissues			
HCC tissue	10	1.30 ± 1.77	0.001
Adjacent tissue	10	3.99 ± 0.77	

### *RBP4* mRNA expression and survival analysis in UALCAN

The mRNA expression levels of *RBP4* between the HCC tissue and normal liver tissue were analysed using UALCAN database. UALCAN (http://ualcan.path.uab.edu/index.html) was a web‐based tool, which allowed researchers to perform analyses of TCGA gene expression data [18]. In addition to gene expression analysis, we performed survival analysis in UALCAN. The HCC patients were divided into high and low expression groups according to the expression of RBP4. The overall survival of HCC patients was analysed using a Kaplan–Meier plot. Log rank *P* value was calculated and displayed on the web page.

### Statistical analysis

ImageJ image software was used to evaluate the grey value of western blot bands and Coomassie brilliant blue staining band. IBM SPSS Statistics 22.0 (IBM Corp, Armonk, NY, USA) was used for statistical analysis. The differential expression of RBP4 between HCC and adjacent non‐tumorous liver tissues was determined using the two‐tailed paired Student’s *t*‐test. One‐way ANOVA was used to analyse the differences amongst multiple groups. *P* < 0.05 was considered statistically significant. Origin 9.1 was used to calculate the antibody concentration corresponding to the EC50 of the mAb.

## Results

### *RBP4* gene cloning and recombination

**Fig. 1:**
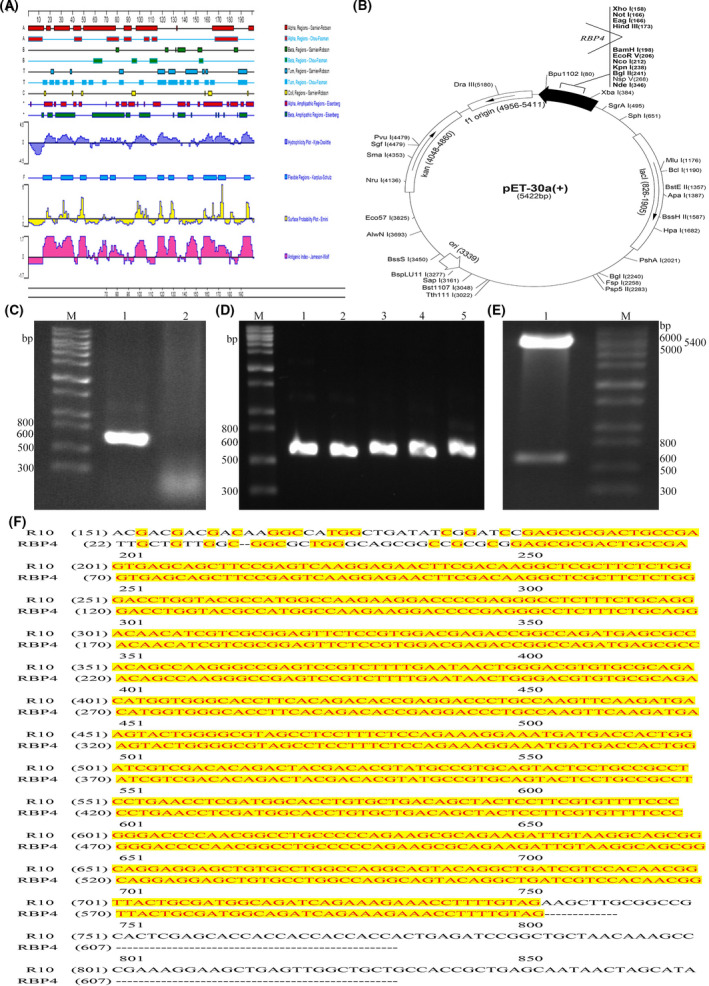
*RBP4* gene cloning and recombination. (A) The immunogenicity of RBP4 was predicted by DNAstart, the immunogenicity of the RBP4 was mainly predicted based on the surface possibility, flexibility, hydrophilicity and antigenic index, etc. (B) Construction of the pET30a‐*RBP4* expression vector. The *RBP4* gene was inserted between BamH I and Hind III sites. (C) RT‐PCR amplification product analysis of RBP4 gene, M: DNA marker, 1: *RBP4* amplification product, 2: negative control. (D) RT‐PCR identification of monoclonal colonies, M: DNA marker, 1 ~ 5: pET30a‐*RBP4* recombinant bacteria verification product. (E) Identification of recombinant plasmid by double‐restriction enzyme digestion, M: DNA marker, 1: pET30a‐*RBP4* double digestion product. (F) Sequence alignment result of recombinant plasmid, R10: Sequence of the positive clonal bacterial solution, RBP4: Sequence of RBP4 gene (NCBI Gene ID: 5950). Data are expressed as the three experiments.

According to the prediction of DNA start software, the surface possibility and flexibility regions of RBP4 protein were moderate, indicating a higher possibility of forming B‐cell epitope and easy to bind to antibodies. The index score of 19‐201aa sequence RBP4 protein antigen, as an immunogen, was moderate, and the local hydrophilicity was favourable, thereby producing a stronger immune response (Fig. 1A). SignalP5.0 analysis showed that 1‐18aa was the signal peptide sequence. The 19‐201aa fragment was selected for RBP4 expression to increase the expression level. At the same time, pET30a was selected as the expression vector (Fig. 1B), and the *RBP4* gene was cloned into pET30a. Specific primers were used to amplify the *RBP4* gene fragment with a size of ~ 600 bp, and the extracted cDNA of human normal liver cell line HL‐7702 was used as a template (Fig. 1C). Five recombinant strains were selected for RT‐PCR amplification during the transformation to obtain the RBP4 gene fragment, and electrophoresis showed a 600 bp target sequence fragment (Fig. 1D). After the recombinant plasmid pET30a‐*RBP4* was successfully transferred into DH5α competent cells, it was digested with BamH I and Hind III enzymes. Finally, pET30a plasmid at 5400 bp and *RBP4* gene fragment at 600 bp were obtained (Fig. 1E). The pET30a‐*RBP4* clonal bacterial solution was sent for sequencing, and the result was consistent with the gene coding sequence published by NCBI (Fig. 1F). This finding indicates that the vector had been successfully constructed and can be used for subsequent experiments.

### Expression and purification of RBP4 recombinant protein

**Fig. 2:**
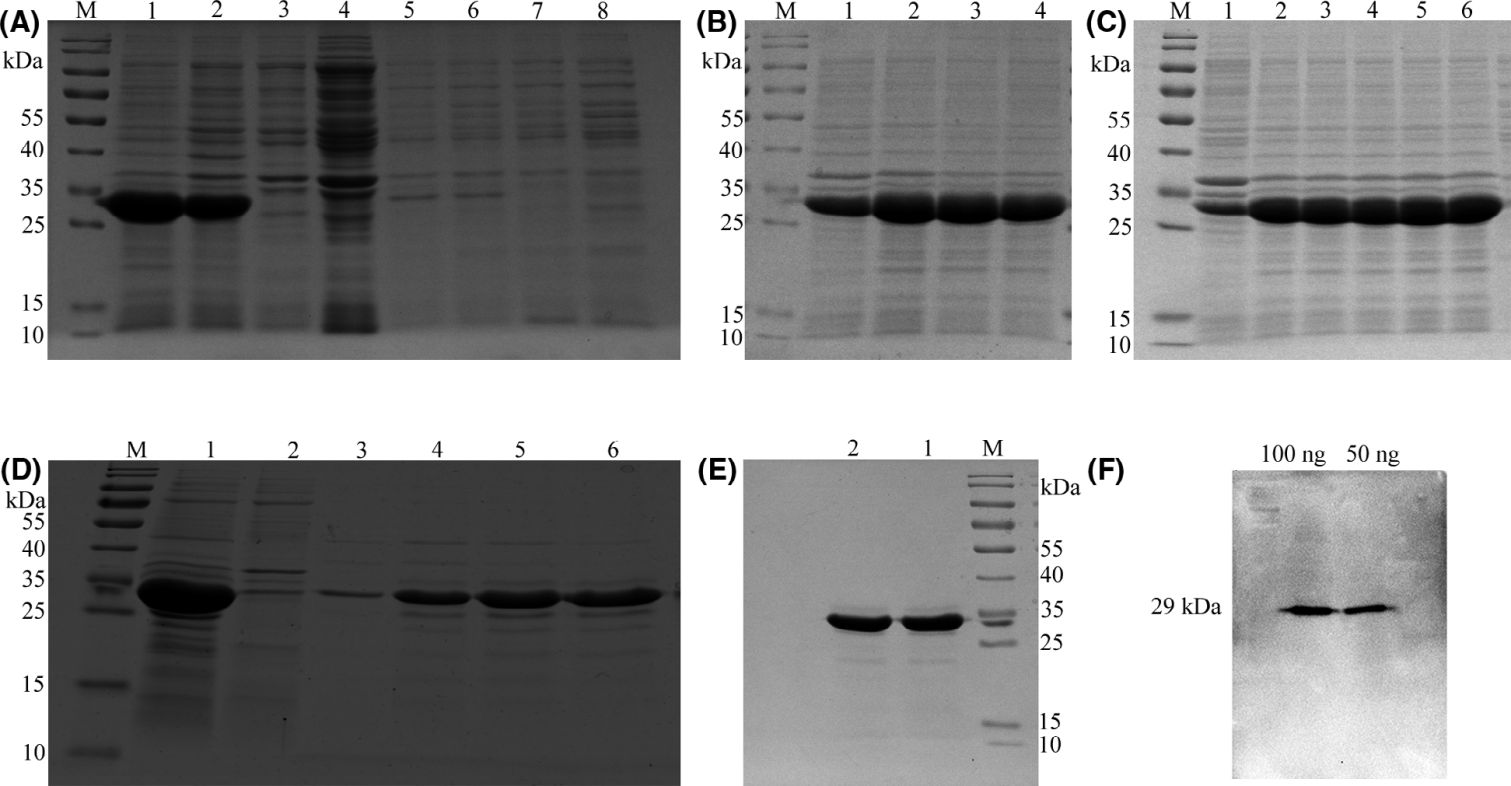
RBP4 recombinant protein expression, purification and identification. (A) Determination of recombinant protein expression form, M: protein marker, 1: precipitate of the pET30a‐*RBP4* group with IPTG induction, 2: precipitate of the pET30a‐*RBP4* group without IPTG induction, 3: precipitate of the pET30a group with IPTG induction, 4: precipitate of the pET30a group without IPTG induction, 5: supernatant of the pET30a‐*RBP4* group with IPTG induction, 6: supernatant of the pET30a‐*RBP4* group without IPTG induction, 7: supernatant of the pET30a group with IPTG induction, 8: supernatant of the pET30a group without IPTG induction. (B) The influence of different temperatures, M: protein marker, 1: 18 °C, 2: 28 °C, 3: 37 °C, 4: 42 °C. (C) The influence of different IPTG concentrations, M: protein marker, 1: 0 mm, 2: 0.2 mm, 3: 0.4 mm, 4: 0.6 mm, 5: 0.8 mm, 6: 1.0 mm IPTG. (D) Ni column purification analysis of RBP4 recombinant protein, M: protein marker, 1: initial protein solution, 2: upper column effluent, 3: 50 mm imidazole, 4: 100 mm imidazole, 5: 150 mm imidazole, 6: 200 mm imidazole. (E) Purification of RBP4 recombinant protein, M: protein marker, 1 ~ 2: the duplicate for purified RBP4 recombinant protein. (F) Western blot identification of RBP4 recombinant protein. Data are expressed as the three experiments.

After conditional exploration, the RBP4 recombinant protein was highly expressed in the precipitated inclusion body, and bands at approximately 29 kDa (RBP4‐His) were evident in lanes 1 and 2, indicating that the *E. coli* BL21 was successfully induced to express the RBP4 recombinant protein (Fig. 2A). The proportion of RBP4 recombinant protein to total protein was 73.99%. The results of induction of recombinant protein expression at different induction temperatures (18, 28, 37 and 42 °C) showed that the optimal induction temperature for RBP4 recombinant protein was 28 °C (*P* < 0.000) (Fig. 2B). Furthermore, the expression level of RBP4 recombinant protein was the highest when IPTG was 1.0 mm (*P* < 0.000) (Fig. 2C). Under optimal induction conditions, RBP4 recombinant protein accounted for 92.06% of the total protein. The RBP4 recombinant protein was preliminarily purified by the Ni column. According to the elution results, the RBP4 recombinant protein was gradually eluted with imidazole at concentrations of 100, 150 and 200 mm, and a higher concentration of RBP4 recombinant protein was found in imidazole with 150 and 200 mm (Fig. 2D). The RBP4 recombinant protein was purified by the Ni column, followed by the KCl gel‐cutting recovery method. The RBP4 recombinant protein was obtained with a purity of 98% by SDS/PAGE electrophoresis, and the protein size was consistent with the expectation (Fig. 2E). The purified RBP4 protein was transferred to PVDF membrane, and the anti‐His antibody was used as the primary antibody. Western blot analysis showed that the RBP4 recombinant protein with His tag could specifically bind to the anti‐His antibody, and the band deepened with the increase in the sample loading amount of RBP4 protein (Fig. 2F).

### Preparation and purification of the RBP4 mAb

**Fig. 3:**
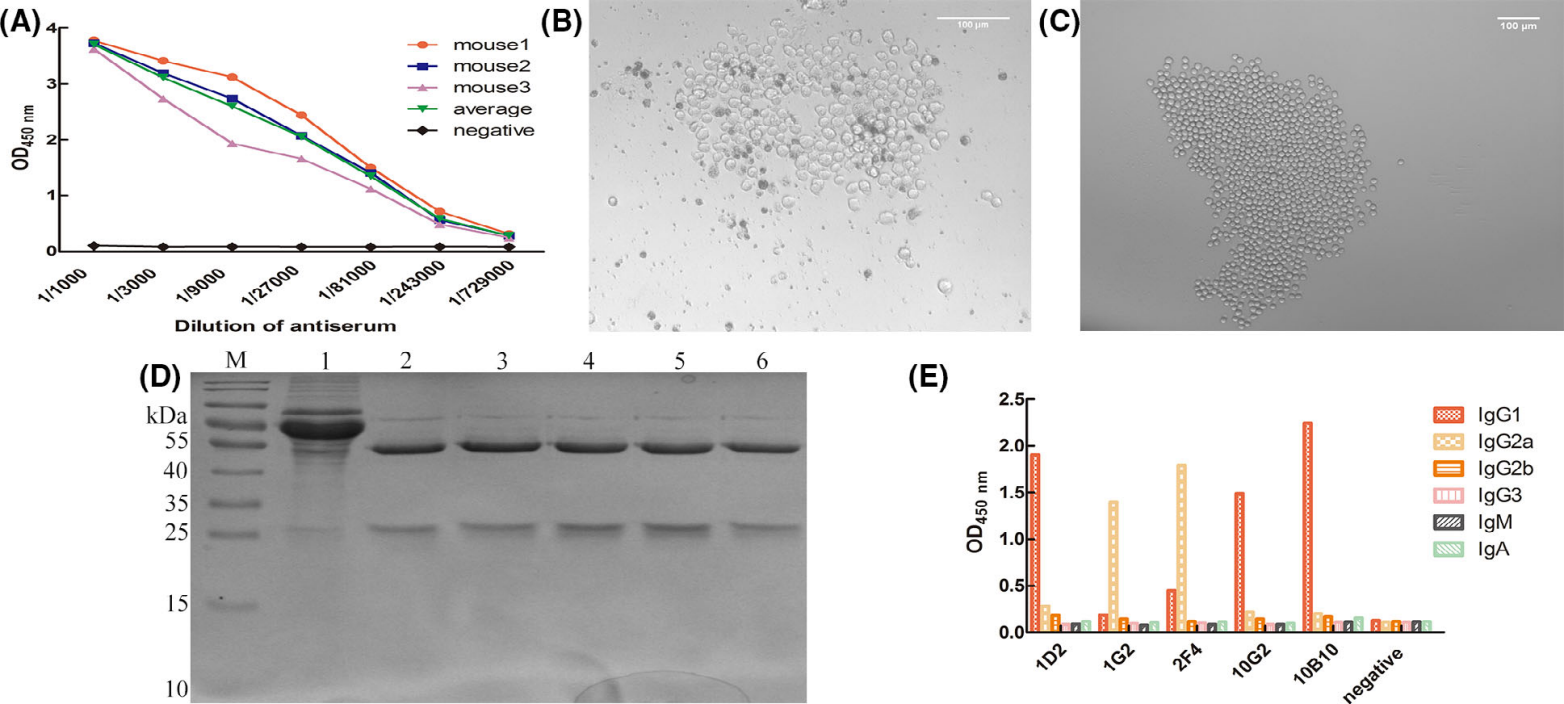
Preparation and purification of RBP4 mAb. (A) Serum antibody titre of mouse after immunisation with RBP4 protein was tested by iELISA. Data are expressed as the mean ± SD of three experiments. (B) Screening of hybridoma cells in 1/3 HAT medium. Hybridoma cells were observed after 8 days of fusion; the scale bar represents 100 μm. (C) Screening and subcloning of hybridoma cells in 20% HT medium. Hybridoma cells were observed after 8 days of subcloning; the scale bar represents 100 μm. (D) mAb ascites purification, M: protein marker, 1: ascites, 2: 1D2, 3: 1G2, 4: 2F4, 5: 10B10, 6: 10G2. (E) mAb subtype determination.

After immunising the mice with purified RBP4 recombinant protein as the immunogen, the serum titre was determined by iELISA. The polyclonal antibody serum titre of three mice could reach 1 : 729,000 (Fig. 3A). The average P/N of the anti‐RBP4 polyclonal antibody was 3.9 (ratio > 2.1), which indicated that the purified RBP4 recombinant protein obtained by this method still had an ideal immunogenicity and could be used for the next step of mAb preparation. After fusion of mouse spleen cells and myeloma cells, hybridoma cells were selected, considering the medium containing 1/3 HAT (Fig. 3B). After two cell fusion experiments, the cell fusion rates were 14.79% and 25.37%. However, no positive hybridioma strains were screened out by iELISA. Amongst nine 96‐well plates, the cell fusion rate of the third sample was 90%, and 32 positive strains were screened out by determining the titre of hybridoma cell supernatant, and the positive rate was 5.00%. The hybridoma cells were subcloned by the limiting dilution method, and the hybridoma cells after subcloning for 8 days showed a single‐cell cluster growth morphology (Fig. 3C). Then, the hybridoma cells with more vigorous growth and single‐cell cluster growth morphology were selected for the second subcloning. After subcloning for three times, all cell lines in the 96‐well plate were positive, and the test result of the hybridoma cell 1D2 is shown in Table 2. Finally, five monoclonal hybridoma cell lines stably secreting anti‐RBP4 mAb, namely 1D2, 1G2, 2F4, 10G2 and 10B10, were obtained. The five monoclonal hybridoma cell lines were expanded in large quantities by ascites induction method. The antibody was purified according to the experimental method of rProtein G affinity chromatography column, and the purity of the antibody was tested by SDS/PAGE gel electrophoresis. The results showed that the antibodies of the five monoclonal hybridoma cell lines showed evident bands at 55 kDa (heavy chain) and 25 kDa (light chain). In addition, no other miscellaneous bands were found on the gel. This finding showed that the mAb obtained in this experiment has high purity (Fig. 3D).

**Table 2:** The hybridoma cell 1D2 96‐well plate of OD_450nm_ value. a, positive control b, negative control.

	1	2	3	5	6	7	8	9	10	11	12
A	1.13	1.39	1.20	1.30	1.27	1.45	1.29	1.10	1.04	1.07	2.17^a^
B	1.19	1.26	1.26	1.19	1.15	1.27	1.18	1.16	1.07	1.22	1.01
C	1.28	1.29	1.23	1.21	1.17	1.21	1.18	1.19	1.08	1.12	1.04
D	1.13	1.15	1.05	1.12	1.06	1.10	1.03	1.02	1.00	1.12	1.00
E	1.17	1.16	1.04	1.08	1.12	1.13	1.08	1.05	0.98	1.06	0.96
F	1.46	1.22	1.27	1.27	1.27	1.22	1.37	1.26	1.15	1.17	1.05
G	1.32	1.29	1.23	1.28	1.22	1.19	1.16	1.24	1.20	1.39	0.97
H	1.37	1.57	1.31	1.26	1.31	1.33	1.65	1.31	1.25	1.36	0.05^b^

### Identification of RBP4 mAb

**Fig. 4:**
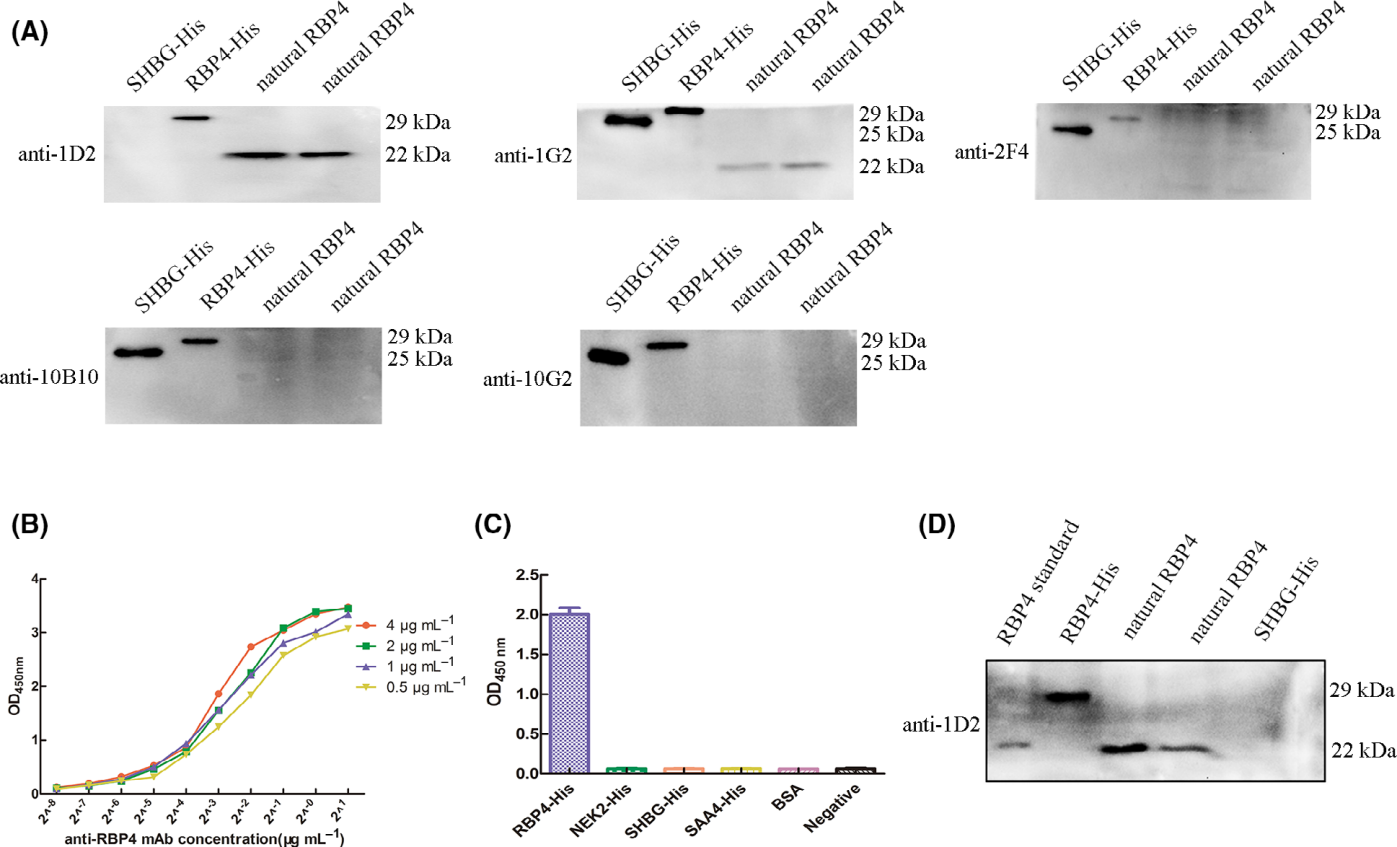
Identification of RBP4 mAb. (A) Determination of monoclonal strains specificity. (B) mAb affinity determination. (C) iELISA for mAb specificity determination, data were described as means ± SD of three experiments. (D) Western blot for mAb specificity determination.

The antibodies of the five purified monoclonal hybridoma cell lines were identified according to the experimental method of the subtype kit. The results showed that the five monoclonal antibodies belonged to different subtypes of IgG (Fig. 3E). Western blot was used to distinguish the specificity of monoclonal antibodies secreted by the five monoclonal strains. The results showed that the 1D2 mAb could react with the recombinant RBP4‐His protein expressed in prokaryotic expression system and the natural RBP4 protein expressed by HCC cells Hep3B and Huh7. However, no cross‐reaction was observed with the recombinant SHBG‐His control. Other monoclonal strains could cross‐react with the recombinant SHBG‐His control (Fig. 4A), indicating that the 1D2 mAb had a higher specificity against RBP4. Therefore, 1D2 was selected for further experiments. The affinity constant of purified 1D2 mAb was determined by iELISA. The EC50 of the antibody with the concentration of 0.5, 1, 2 and 4 µg·mL^−1^ RBP4 recombinant protein was 0.197, 0.154, 0.166 and 0.122 µg·mL^−1^, respectively (Fig. 4B). Therefore, the average affinity constant of anti‐RBP4 mAb was 3.98 × 10^8^ L·mol^−1^. Recombinant RBP4‐His protein, recombinant SHBG‐His protein, recombinant SAA4‐His protein, recombinant NEK2‐His protein, BSA albumin and diluent negative control were coated on 96‐well ELISA plate, and the specificity of the anti‐RBP4 mAb was identified by iELISA. The anti‐RBP4 mAb only specifically reacted with recombinant RBP4‐His protein, and it did not cross‐react with other recombinant proteins containing His tag, BSA albumin or diluent negative control (Fig. 4C). The western blot result also showed that the anti‐RBP4 mAb interacted with natural RBP4 protein expressed by HCC cells Hep3B and Huh7 and illustrated the same position as the standard RBP4 protein (R&D). However, the recombinant SHBG‐His protein did not cross‐react, indicating that the anti‐RBP4 mAb only and specifically bound to the RBP4 protein (Fig. 4D).

### Lower expression of RBP4 in HCC tissues by immunohistochemical detection with anti‐RBP4 mAb

**Fig. 5:**
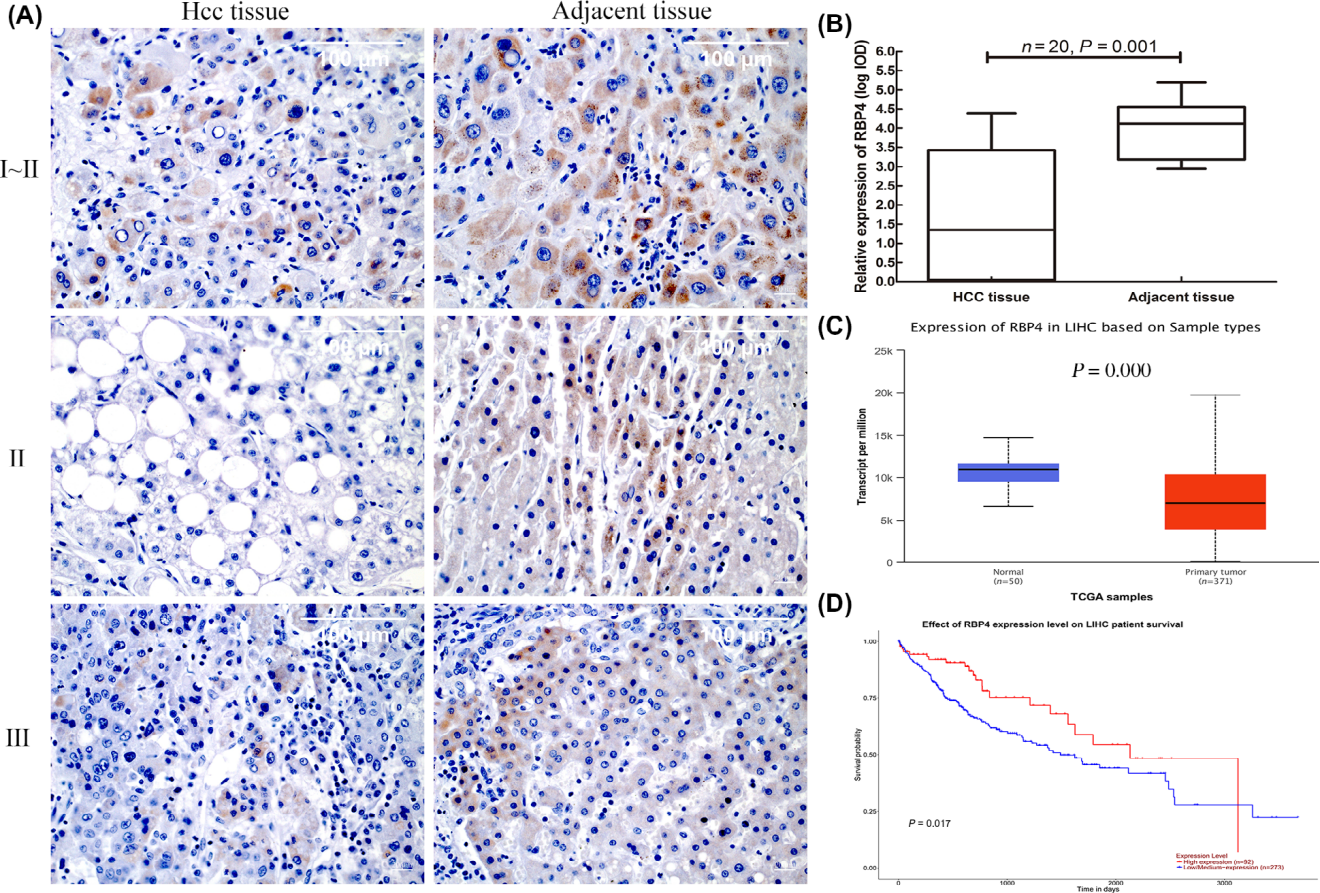
Immunohistochemical staining of HCC tissue and adjacent tissue with anti‐RBP4 mAb and survival analysis. (A) Immunohistochemical staining for RBP4 expression in HCC tissues and adjacent non‐tumorous liver tissues; the scale bar represents 100 μm. Data are expressed as the mean ± SD of three experiments (B) Immunohistochemical staining integrated optical density (IOD) value of RBP4 in HCC tissues and adjacent non‐tumorous liver tissues (*n* = 20 tissue samples, *P* = 0.001), data were analysed using the two‐tailed paired Student’s *t*‐test. (C) The mRNA expression of RBP4 in HCC versus normal was analysed using the UALCAN web tool, the mRNA expression data were analysed based on TCGA database. (D) The prognostic value of the RBP4 expression in the HCC patients was analysed using the UALCAN web tool the prognostic value was analysed based on TCGA database.

The expression of RBP4 in 10 pairs of HCC tissues and adjacent non‐tumorous liver tissues was detected by immunohistochemical method with anti‐RBP4 mAb to explore the value of anti‐RBP4 mAb in the HCC. The result indicated that anti‐RBP4 mAb detected the expression of RBP4 protein in HCC tissues and adjacent tissues of different pathological grades (Fig. 5A). image‐pro Plus 6.0 digital image analysis software was used to semi‐quantitatively analyse the positive reaction of brown colour. We found that RBP4 expression was strongly positively stained on adjacent non‐tumorous liver tissues and weakly positively stained on HCC tissues. The RBP4 expression in HCC tissues was significantly lower than in adjacent non‐tumorous liver tissues (*P = *0.001) (Fig. 5B). This finding was consistent with the results of The Cancer Genome Atlas (TCGA) database (Fig. 5C). At the same time, to evaluate the clinical significance of RBP4 expression in HCC patients, Kaplan–Meier survival analysis of the expression level of RBP4 in HCC patients from TCGA database was conducted based on UALCAN. The results showed that HCC patients with lower RBP4 expression had poorer prognosis (log‐rank test, *P* = 0.017) (Fig. 5D).

## Discussion

RBP4 protein, as a secreted protein of the retinol family, is widely distributed in blood, urine and other body fluids. The normal concentration of human serum RBP4 protein is 25–70 mg·L^−1^, and the urine concentration is 0.7 mg·L^−1^. Serum RBP4 transports retinoid proteins to target organs and reabsorbs them back into the blood through renal tubules. Therefore, RBP4 is often used as a clinical indicator for early diagnosis and efficacy evaluation of renal diseases [19]. Liver is the main organ for RBP4 protein synthesis; thus, its health status greatly affects the expression level of RBP4 [20]. Some studies have shown that the serum RBP4 expression level of patients with chronic hepatitis B infection is lower than that of healthy people, and it is negatively correlated with the severity of the disease [21]. The detection of serum retinol and prealbumin levels in patients with liver cirrhosis secondary to hepatitis may represent a sensitive indicator of acute liver damage. RBP4, as an adipokine, is closely related to the severity of liver disease in patients with alcoholic cirrhosis [22]. Researches have found that the concentration of serum RBP4 is negatively correlated with the disease severity in patients with early HCV liver fibrosis, and its concentration decreases as the degree of liver fibrosis increases [12]. The negative correlation between RBP4 and the severity of liver fibrosis might be due to the decreased level of RBP4, which participated in the activation of hepatic stellate cell overexpression and the deposition of type I collagen in the liver, that promoted the progression of liver fibrosis [12, 23], which eventually developed into liver cirrhosis or HCC. In summary, changing the concentration of serum RBP4 might represent a sensitive indicator of liver function injury.

In the clinical testing, antibodies are the commonly used tools. At present, reports on the preparation of monoclonal antibodies against RBP4 are few. The RBP4 protein was analysed by bioinformatics analysis, and the N‐terminal amino acid sequence (1‐18aa) of the RBP4 protein was found to be the secretion signal peptide sequence. Thus, the amino acid sequence with favourable epitope specificity (19‐201aa) was selected for the protein expression. *E. coli* was selected as a protein expression tool for expressing RBP4 recombinant protein, considering its characteristics of prokaryotic expression system with clear genetic background, strong operability, mature and stable technology, large yield and low cost [24, 25]. After reasonable IPTG inducers and optimised temperature expression conditions, the proportion of RBP4 recombinant protein in the total protein was increased, and the yield of RBP4 expressed in Escherichia coli was effectively increased. The inclusion body recombinant protein should be renatured with a certain concentration of urea because the RBP4 recombinant protein existed in the form of precipitated inclusion bodies. Thus, the inactive recombinant protein could be folded accurately to restore its activity and maintain appropriate antigen activity [26, 27]. The findings indicated that the imidazole concentration of 150 mm was beneficial to the efficient recovery of the target protein, but still a small amount of other protein was found, probably affecting the effect of RBP4 protein as an immunogen. After the initial purification by the Ni affinity chromatography column, RBP4 recombinant protein was further purified by KCl silver‐stained gum recovery to obtain a higher purity RBP4 recombinant protein. During the gum recovery process, the imidazole, urea and salt ions in the RBP4 recombinant protein solution were also removed, reducing the loss of the recombinant protein in the final dialysis process, which was conducive to obtaining a large amount of high‐purity recombinant protein. The RBP4 recombinant protein was successfully identified by western blot, indicating that the recombinant protein expressed in the form of inclusion bodies maintained the integrity of the linear epitope and had compelling antigenicity after renaturation.

The results of serum titre showed that the serum antibody titres of mice could reach more than 1 : 729,000, which was much higher than the currently reported literature [28, 29]. This finding was attributed to the use of high‐purity RBP4 recombinant protein as the immunogen and the low‐dose, long‐term immunisation procedure to induce the production of anti‐RBP4 antibodies in mice, avoiding high‐dose injection of immunogen to cause immune tolerance in mice. Therefore, the high‐purity RBP4 recombinant protein was obtained as an antigen with better immune response effect, reducing the production of nonspecific antibodies. In addition a low‐dose, long‐term immunisation procedure could effectively induce high‐titre serum titres to improve the efficiency of screening high‐affinity and high‐specificity monoclonal antibodies. After three cell fusion experiments, several aspects should be considered in the preparation of positive hybridoma cell lines. Firstly, the dose of immunogen injected into the intraperitoneal cavity should be sufficiently large to stimulate the mouse spleen to differentiate into more antibody‐secreting splenocytes. Secondly, myeloma cells should be well maintained, and the fusion ratio of the mouse spleen cells and myeloma cells should be as appropriate as possible. Finally, in the cell fusion process, control of the action time on PEG solution was beneficial to increase the fusion rate; preheating of the medium reduces cell membrane damage, and gentle action during the entire operation process would be beneficial to the growth and survival of hybridoma cells.

Low‐dose and long‐term immunisation programme was conducted, and the antibodies secreted by the five monoclonal hybridoma cell lines were demonstrated to be IgG subtypes, which could bind to antigens stably and effectively. They are often adopted as stable and widely used monoclonal hybridioma subtypes. Our research results also showed that the average affinity constant of RBP4 mAb was 3.98 × 10^8^ L·mol^−1^. The affinity constant of mAb was referred to as the specific binding strength of the antigenic determinant and the antibody. Higher affinity was associated with stronger specificity. When the affinity constant was between 10^7^ and 10^12^ L·mol^−1^, it belonged to the high‐affinity antibody [30], and the high‐affinity mAb had a wide range of application value. At the same time, the specificity of anti‐RBP4 mAb was determined by iELISA and western blot; it indicated that the anti‐RBP4 mAb did not produce nonspecific reaction. The recombinant RBP4 protein expressed in the prokaryotic expression system, RBP4 protein standard (R&D) expressed in the eukaryotic expression system and natural RBP4 protein secreted by HCC cell lines could all react specifically and were more conducive to the accurate detection of RBP4, effectively avoiding false‐positive results.

The TCGA database contains comprehensive cancer genome data, including mutations, copy number variations, mRNA expression, miRNA expression and methylation data [31]. TCGA is widely recognised amongst most researchers and has been widely used to verify the consistency of experimental data [32]. The immunohistochemical analysis showed that the expression level of RBP4 protein in HCC tissues was significantly lower than the adjacent tissues, and it decreased with the increase in HCC pathological grade. These results were highly consistent with the analysis results of the TCGA database. In addition, Kaplan–Meier survival curve analysis showed that low RBP4 expression in HCC patients had a poor prognosis, suggesting that RBP4 may represent a potential prognostic factor in HCC patients. Therefore, anti‐RBP4 mAb has potential application value for immunohistochemical detection.

The liver is the main secretion site of RBP4 protein. When it is damaged, it affects the synthesis of RBP4, resulting in the decrease in serum RBP4 concentration, accompanied by the aggravation of liver damage. With the deepening of studies, researchers found that the expression level of RBP4 was closely related to cancers. In head and neck cancer [33], breast cancer [34], hepatocellular carcinoma [11] and other cancers, serum RBP4 levels were significantly reduced, and the decrease might be caused by methylation [35]. Therefore, the decrease in RBP4 expression level in cancer tissues might be a new indicator for early detection of cancers in the clinic. This finding indicates that detecting the expression level of RBP4 in liver tissue might have diagnostic significance for early detection of HCC.

However, the current study also had several shortcomings in the process of preparing monoclonal antibodies. For example, only two types of antibodies secreted by the newly prepared five monoclonal hybridoma cell lines could be used for detection by western blot and immunohistochemistry simultaneously, and the three remaining strains could only be used for immunohistochemical detection. This finding might be because the anti‐RBP4 monoclonal antibodies secreted by the three strains specifically recognised the internal conformational epitopes of natural RBP4 protein, rather than the linear epitopes on the protein surface. As for the application of anti‐RBP4 mAb in immunohistochemical detection, we will consider to expand the sample size to detect HCC patients at different pathological grades in the future. In addition, the application value of anti‐RBP4 mAb in the diagnosis of HCC will be evaluated.

## Conclusions

In conclusion, the recombinant RBP4 protein with high concentration, high purity (98%) and outstanding antigenic activity was successfully expressed and obtained. The expression and purification method had important reference value for the development of a large‐scale standardised production process of RBP4 recombinant protein. The anti‐RBP4 mAb was prepared; it had high affinity and strong specificity and could specifically bind to the natural RBP4 protein and suitable for immunohistochemical analysis.

## Conflict of interest

The authors declare no conflict of interest.

## Data accessibility

All data in our study are available from the corresponding author on reasonable request.

## Author contributions

HL performed the experiment, analysed data and wrote the original draft. WS, LCY, QLC, SPH performed the animal experiment. XH, YSL, FJW analysed the data and revised the manuscript. MH designed the study, supervised experiments, MH and XH supported the study. All authors reviewed the final manuscript.
